# Physicochemical and Antimicrobial Characterization of Beeswax–Starch Food-Grade Nanoemulsions Incorporating Natural Antimicrobials

**DOI:** 10.3390/ijms18122712

**Published:** 2017-12-15

**Authors:** Teresita Arredondo-Ochoa, Blanca E. García-Almendárez, Monserrat Escamilla-García, Olga Martín-Belloso, Giovanna Rossi-Márquez, Luis Medina-Torres, Carlos Regalado-González

**Affiliations:** 1 DIPA, PROPAC, Facultad de Química, Universidad Autónoma de Querétaro. C.U., Cerro de las Campanas s/n, Col. Las Campanas, Querétaro 76010, Qro., Mexico; arredondo.tere@yahoo.com (T.A.-O.); blancag31@gmail.com (B.E.G.-A.); moneg14@hotmail.com (M.E.-G.); 2 Department of Food Technology, University of Lleida–Agrotecnio Center, Avda. Alcalde Rovira Roure, 191, E-25198 Lleida, Spain; omartin@tecal.udl.cat; 3 Instituto Tecnológico “José Mario Molina Pasquel y Henríquez”–Unidad Académica Lagos de Moreno, Libramiento Tecnológico No. 5000, Col. Portugalejo de los Romanes, C.P., Lagos de Moreno 47480, Jalisco, Mexico; gio_rossi@yahoo.com; 4 Facultad de Química, Área de Reología de Materiales Complejos, Universidad Nacional Autónoma de México, Circuito Interior s/n, CDMX 04510, Mexico; luismt@unam.mx

**Keywords:** nanoemulsion, oxidized corn starch, beeswax, antimicrobials

## Abstract

Nanoemulsions are feasible delivery systems of lipophilic compounds, showing potential as edible coatings with enhanced functional properties. The aim of this work was to study the effect of emulsifier type (stearic acid (SA), Tween 80 (T80) or Tween 80/Span 60 (T80/S60)) and emulsification process (homogenization, ultrasound or microfluidization) on nanoemulsion formation based on oxidized corn starch, beeswax (BW) and natural antimicrobials (lauric arginate and natamycin). The response variables were physicochemical properties, rheological behavior, wettability and antimicrobial activity of BW–starch nanoemulsions (BW–SN). The BW–SN emulsified using T80 and microfluidized showed the lowest droplet size (77.6 ± 6.2 nm), a polydispersion index of 0.4 ± 0.0 and whiteness index (WI) of 31.8 ± 0.8. This BW–SN exhibited a more negative ζ-potential: −36 ± 4 mV, and Newtonian flow behavior, indicating great stability. BW–SN antimicrobial activity was not affected by microfluidization nor the presence of T80, showing inhibition of the deteriorative fungi *R. stolonifer*, *C. gloeosporioides* and *B. cinerea*, and the pathogenic bacterium *S.* Saintpaul. In addition, regardless of emulsifier type and emulsification process, BW–SN applied on the tomato surface exhibited low contact angles (38.5° to 48.6°), resulting in efficient wettability (−7.0 mN/m to −8.9 mN/m). These nanoemulsions may be useful to produce edible coatings to preserve fresh-produce quality and safety.

## 1. Introduction

The food industry is consistently looking for innovative technologies to improve quality, safety and functional properties of food products [1]. Nanotechnology, a field that involves manipulation of matter at nanometer scale, has a promising potential to produce nanoemulsions that can be used to obtain novel packaging material [2]. The incorporation of hydrophobic agents such as beeswax (BW) into polysaccharides or protein suspensions is a potential food-packaging alternative to prevent water loss and to improve appearance of fresh produce when applied as edible coatings [3,4]. To overcome solubility limitations of lipophilic ingredients, nanoemulsions have been proposed as suitable delivery systems [5]. Oil-in-water (O/W) nanoemulsions consist of small lipid droplets (10–100 nm in diameter) dispersed in an aqueous continuous phase. Their low particle size provides slight turbidity, being suitable for a wide range of food applications [6,7]. In addition, nanoemulsions may enhance the transport of active compounds such as antimicrobials through biological membranes, intensifying their antimicrobial activity [8]. Nevertheless, nanoemulsions are metastable systems because of the surface free energy required to increase the interfacial area between oil and water phases, and tend to break down over time due to gravitational separation and droplet aggregation [9,10]. Emulsifiers adsorb in oil-water interfaces, lowering the interfacial tension and preventing or slowing down particle aggregation by increasing repulsion forces [11,12]. Most emulsifiers are amphiphilic molecules, ranging from proteins, polysaccharides and fatty acids, to ionic or non-ionic surfactants [13]. On the other hand, intense disruptive forces generated by homogenization, sonication or microfluidization can produce nanoemulsions where oil and water phases collapse, leading to lipid droplets [14]. Thus, this study aimed to evaluate the type of emulsifier and the influence of processing parameters on the physicochemical, rheological, wettability and antimicrobial properties of nanoemulsions containing beeswax (BW) and oxidized starch (OS), added with natural antimicrobials.

## 2. Results and Discussion

### 2.1. Thermal Properties 

**Figure 1:**
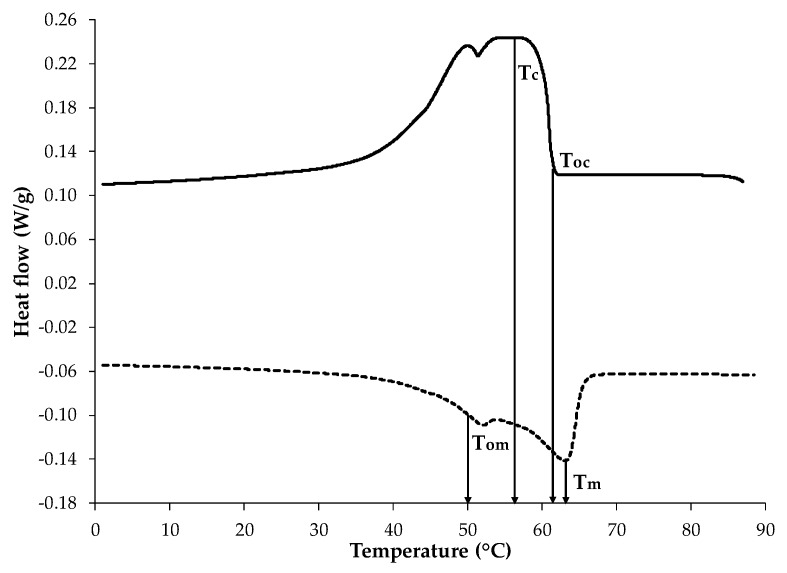
Representative beeswax thermogram: crystallization process (continuous line) and melting process (dotted line), (T_oc_: crystallization onset temperature; T_c_: maximum crystallization peak; T_om_: melting onset temperature; T_m_: maximum melting peak).

The thermal behavior of BW was evaluated through the differential scanning calorimetry (DSC) thermogram (Figure 1), in which the exothermic process was used to estimate the crystallization onset temperature (T_oc_) that was 61.47 ± 0.02 °C. This curve exhibited two peaks, where the dominant crystallization peak (T_c_) was at 57.15 ± 0.09 °C with a crystallization enthalpy (ΔH_c_) of −186.15 ± 1.34 J/g. For the endothermic process, a clear melting onset temperature (T_om_) was observed at 50.64 ± 0.04 °C, showing two peaks, where the maximum melting peak (T_m_) appeared at 63.09 ± 0.06 °C, with a melting enthalpy (ΔH_m_) of 188.85 ± 0.35 J/g. The presence of different peaks from the BW thermal behavior may be attributed to the heterogeneous composition of the BW matrix [15]. These peaks most likely represent the crystallization or melting of distinct BW components; each of them could be single or multicomponent in nature, such as fatty acids (12–14%), monoesters and hydroxymonoesters (35–45%), complex wax esters (15–27%), straight chain hydrocarbons (mainly C33) (12–16%), and other minor components [16,17]. The BW thermal properties obtained in this work are in agreement with those reported by Yilmaz and Ogutou [18] for commercial BW (KahlWax, Kahl GmbH, Trittau, Germany), except for ΔH_c_ (8% smaller) and ΔH_m_ (7.6% smaller), probably associated with different composition. The estimation of BW thermal parameters defined minimal temperature conditions for nanoemulsion formation, to avoid wax solidification.

### 2.2. Physicochemical Characterization of BW–SN

#### 2.2.1. Droplet Size, Polydispersity Index (PDI) and Size Distribution

**Figure 2:**
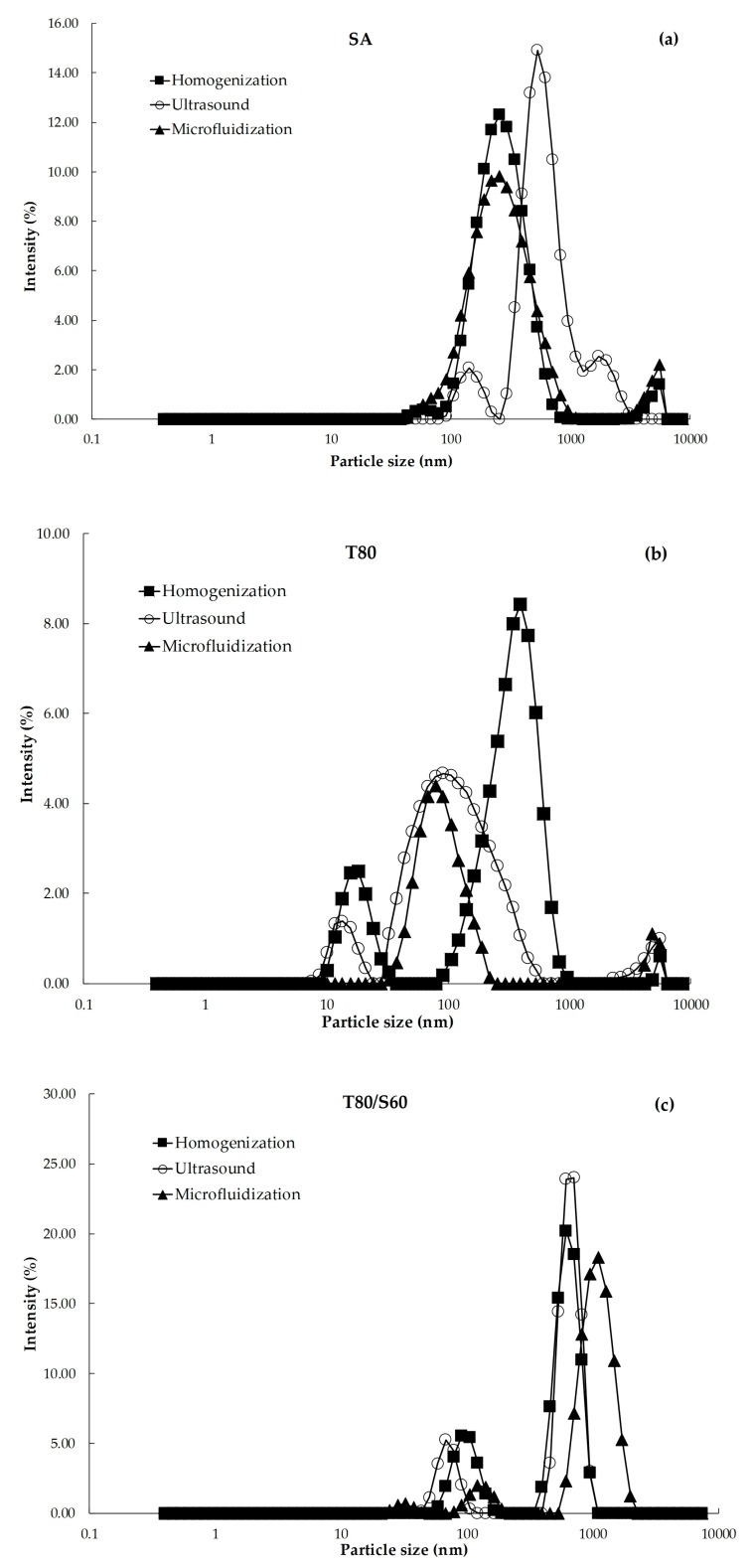
Effect of emulsifier type: (**a**) SA: stearic acid; (**b**) T80: Tween 80; and (**c**) T80/S60: Tween 80/Span 60, and shear process: homogenization, ultrasound and microfluidization, on size distribution of beeswax–starch nanoemulsions.

The dynamic light scattering (DLS) technique assumes that large particles scatter light more strongly than small particles [19], whereas PDI limits vary from 0 to 1 for homogenous and heterogeneous size distributions of emulsions, respectively [20]. Droplet size distributions of the BW–SN (Figure 2) showed several peaks corresponding to BW droplets of different size. Major high-intensity peaks positioned at 1000 nm could be observed in emulsions containing T80/S60 regardless of the emulsification process. However, minor peaks at the nano-range (100 nm) were observed in the BW–SN with T80. According to Rao and McClements [21], the peaks around 10 nm could be associated with emulsifier micelles that were not adsorbed at the oil–water interface of nanoemulsions, whereas residual intensity peaks close to 5000 nm suggest the presence of larger lipid droplets that were not disrupted. A significant interaction (*p* < 0.05) between emulsifier type and emulsification process affected the average droplet size (z-average) and PDI of the BW–SN (Table 1). Using stearic acid (SA) or the mixture T80/S60 as emulsifiers, followed by ultrasound processing, led to the highest droplet sizes (515.1 nm or 1054.3 nm), with PDI values of 0.5 and 0.8, respectively. Conversely, nanoemulsions obtained using T80 as emulsifier and microfluidized led to the smallest droplet size (77.7 ± 6.2 nm) and PDI of 0.3 ± 0.0, which was confirmed by size distribution data (Figure 2). Ghosh et al. [22] reported that low molecular weight non-ionic emulsifiers produce favorable O/W interactions due to their high hydrophilic–lipophilic balance (HLB = 15), decreasing interfacial tension and requiring reduced free energy for nanoemulsion formation. These authors obtained the smallest droplet size of 29.60 ± 0.20 nm with a PDI of 0.21 ± 0.00 for nanoemulsions based on basil oil (6% *v*/*v*) emulsified with T80 in 1:3 (*v*/*v*) ratio. Salvia-Trujillo et al. [23], working with microfluidization, achieved droplet sizes of 7.35 ± 1.67 nm, and close-to-homogeneous nanoemulsion (PDI of 0.34 ± 0.10) for sodium alginate lemongrass oil:T80 in 1:1 (*v*/*v*) ratio using the same conditions as those reported here. The slightly higher droplet sizes obtained in this work were attributed to the low crystallization temperature of BW droplets, tending to coalesce due to their heterogeneous composition.

#### 2.2.2. ζ-Potential

**Figure 3:**
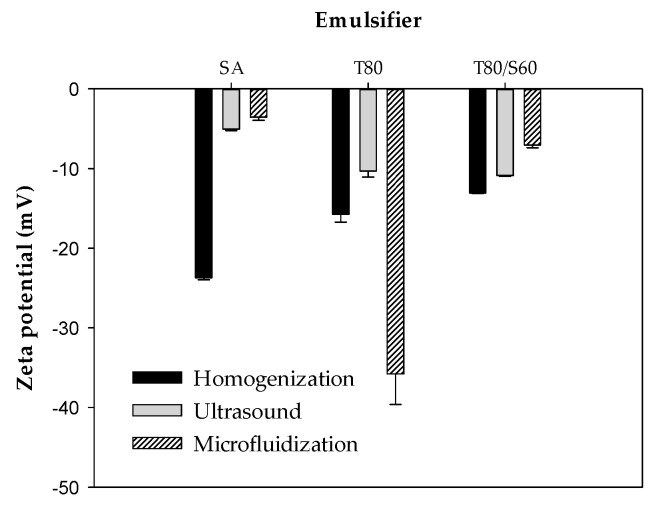
Effect of emulsifier type (SA: stearic acid; T80: Tween 80; T80/S60: Tween 80/Span 60) and shear process (homogenization, ultrasound and microfluidization) on the ζ-potential of beeswax-starch nanoemulsions.

ζ-potential is the difference in electrical charge between the dense layer of ions around the micelle particles and that of the stationary layer of fluid surrounding them [24]. Particles with absolute magnitude of ζ-potential >30 mV are usually considered to be stable, since electrical charge of droplets is strong enough to assume that repulsive forces are predominant in the nanoemulsion system [25]. ζ-potential values of BW–SN (Figure 3) were significantly influenced (*p* < 0.05) by emulsifier type and emulsification processes. Emulsions containing SA subjected to ultrasound, and SA or T80/S60 treated by microfluidization, exhibited the weakest electrical charge of −3.6 ± 0.4 mV, −5.0 ± 0.2 mV and −7.0 ± 0.3 mV, respectively. In contrast, nanoemulsions produced using T80 as emulsifier, and formed by microfluidization, showed the strongest electrical charge of −35.8 ± 3.8 mV. This value is more negative than those reported by Guerra-Rosas et al. [26], ranging from −6.53 mV to −15.20 mV for nanoemulsions based on high-methoxyl pectin and a variety of essential oils, subjected to microfluidization (150 MPa, 5 cycles) with T80 at same oil:emulsifier ratio as in this work. The emulsifier nature controls the surface charge of the BW–SN; thus, lipid droplets stabilized by SA (anionic emulsifier) show negative charge. Furthermore, the negative charge observed in the BW–SN can be attributed to the adsorption of OS molecules in the O/W interface, due to the presence of anionic carboxylic groups [27]. Anionic impurities, such as free fatty acids, in the lipid or emulsifier components can contribute to the observed negative charge [28]. Finally, the use of non-ionic emulsifiers like T80 improves nanoemulsion stability, due to their hydrophilic polyoxyethylene head groups that are able to deposit onto the O/W interface and reduce interfacial tension, protecting lipid droplets against aggregation [29]. The high increase in negative ζ-potential of microfluidized BW–SN emulsified with T80 could be due to its very small droplet size (77.7 ± 6.2 nm), leading to more negatively charged particles.

#### 2.2.3. pH

One of the most important factors that determine the formation and properties of nanoemulsions is the solution pH through ionization of surface groups, and therefore influencing the final surface charge density [30]. In this sense, the pH of the BW–SN showed no significant changes for each surfactant tested regardless of the emulsification process (Table 1). 

#### 2.2.4. Whiteness Index (WI) 

**Figure 4:**
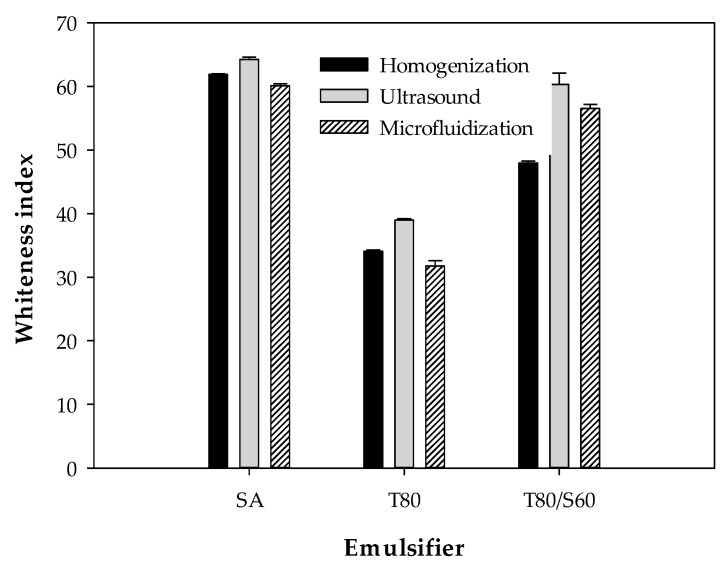
Effect of emulsifier type (SA: stearic acid; T80: Tween 80; T80/S60: Tween 80/Span 60) and shear process (homogenization, ultrasound and microfluidization) on the whiteness index of beeswax–starch nanoemulsions.

A significant interaction (*p* < 0.05) between emulsifier type and emulsification process on the color of the BW–SN, expressed as WI, is shown in Figure 4. Nanoemulsions incorporated with SA or T80/S60, using ultrasound processing, showed the highest WI values of 64.2 ± 0.3 and 60.3 ± 1.7, respectively. In contrast, the WI significantly (*p* < 0.05) improved to 31.7 ± 0.8 using T80 under microfluidization. Salvia-Trujillo et al. [31] confirmed WI reductions through microfluidization at different pressures and cycles, with the lowest WI of 35.71 ± 0.16 at 150 MPa and 3 cycles, for lemongrass oil-sodium alginate emulsified with T80. Similar results were obtained by Guerra-Rosas et al. [26] with WI of 32.94 ± 0.03 for high-methoxyl pectin and thyme essential oil nanoemulsions treated with T80 after microfluidization (150 MPa, 5 cycles). In this way, BW–SNs emulsified with T80 under a microfluidization process might be suitable for development of fresh produce coatings, because of their slightly translucent appearance attributed to nanosized lipid particles, high stability and relatively high homogeneity.

### 2.3. Transmission Electron Microscopy (TEM)

**Figure 5:**
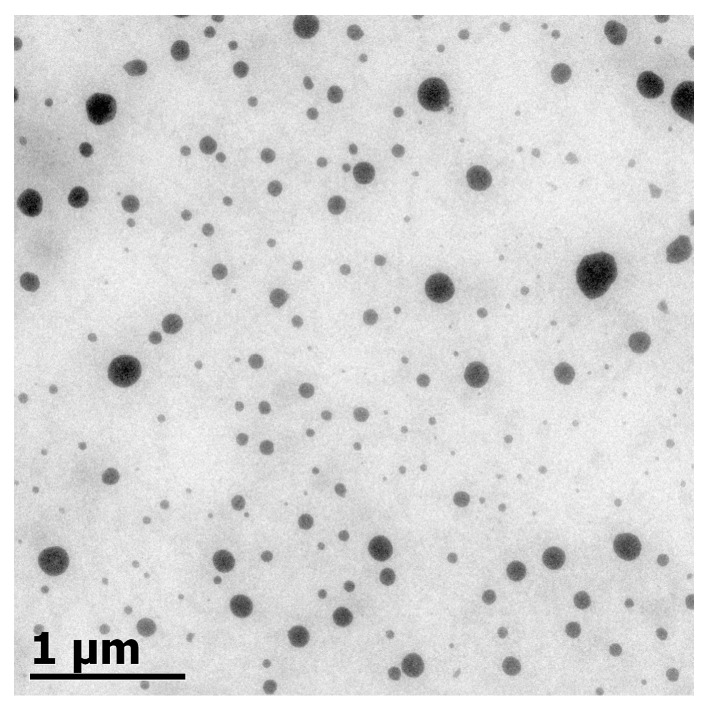
Transmission electron microscopy image of BW–SNs using T80 as emulsifier after microfluidization.

Electron microscopy is an appropriate tool to characterize nanoemulsions since it is able to visualize nanosized structures that cannot be detected by classical microscopy techniques [32]. The average droplet size of estimated BW–SN (Table 1) was confirmed by TEM observations, and for instance, microfluidized BW–SN using T80 as emulsifier showed droplet sizes <100 nm in diameter (Figure 5). In this work, BW–SNs were prepared by a negative-staining technique, and they appear as dark droplets against a white background. However, this may vary according to the affinity of the staining agent to interfacial components [23].

### 2.4. Rheological Characterization

**Figure 6:**
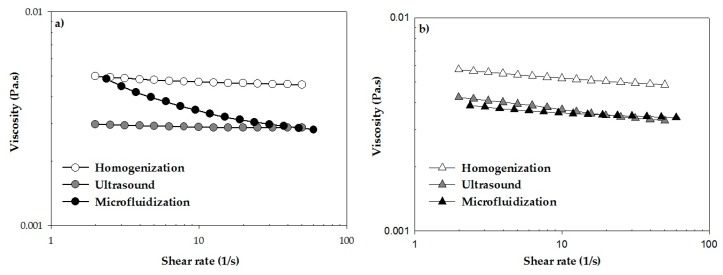

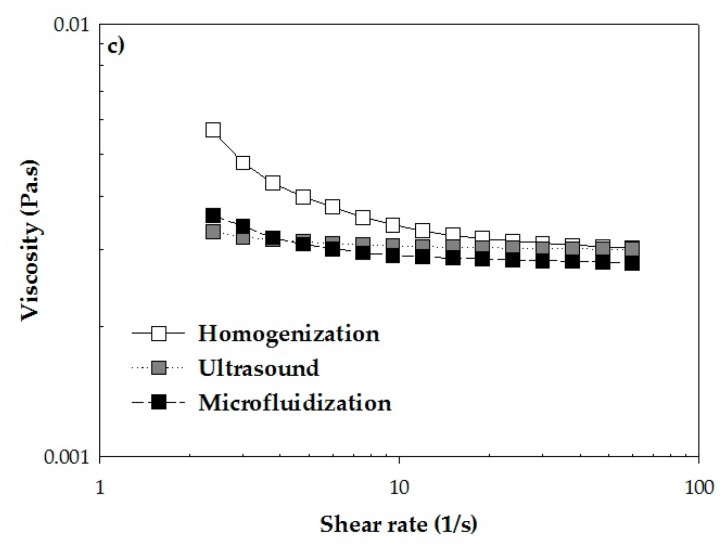
Effect of emulsifier type: (**a**) SA: Stearic acid; (**b**) T80: Tween 80; and (**c**) T80/S60: Tween 80/Span 60, and shear process: homogenization, ultrasound and microfluidization, on rheological behavior of beeswax–starch nanoemulsions.

A significant effect (*p* < 0.05) was observed between the emulsifier type and emulsification process (Figure 6). Dilatant fluids exhibit *n* > 1, whereas pseudoplastic fluids present *n* < 1 [33]. Non-Newtonian “shear-thinning” behavior (*n* < 1) flow curves, associated with agglomeration of starch–wax droplets, was observed for BW–SN added with SA and microfluidized (Figure 6a); T80 subjected to ultrasound (Figure 6b); and T80/S60 homogenized or microfluidized (Figure 6c). According to Hopkins et al. [34], this behavior is likely due to the disruption of interactions (e.g., hydrogen bonding and hydrophobic interactions) between nanoemulsion components (e.g., the starch and BW) when subjected to shearing, producing weaker interactions and greater alignment within the shear field as shear rates increase. These authors reported similar shear-thinning behavior for nanoemulsions based on soy protein isolate and flaxseed oil (3% or 10% *w*/*w*) using T80 (1:0.5 oil-emulsifier (*w*/*w*) ratio) after homogenization; all samples displayed 0.81 ≤ *n* ≤ 0.95. In another study, Rezvani et al. [35] reported a shear thinning behavior for sodium caseinate nanoemulsions and corn oil (1.5% *w*/*w*) emulsified with SA (1% and 2% *w*/*w*) using two homogenization cycles, achieving *n* = 0.68–0.78. The remaining BW–SN presented a Newtonian behavior (*n* = 1), that was attributed to the high amount of continuous phase involved in BW–SN interactions and the low particle size (Table 1). In contrast to our results, Fabra et al. [36] reported that the shear-thinning effect was much stronger in smaller-sized nanoemulsions.

#### Viscoelastic Properties

**Figure 7:**
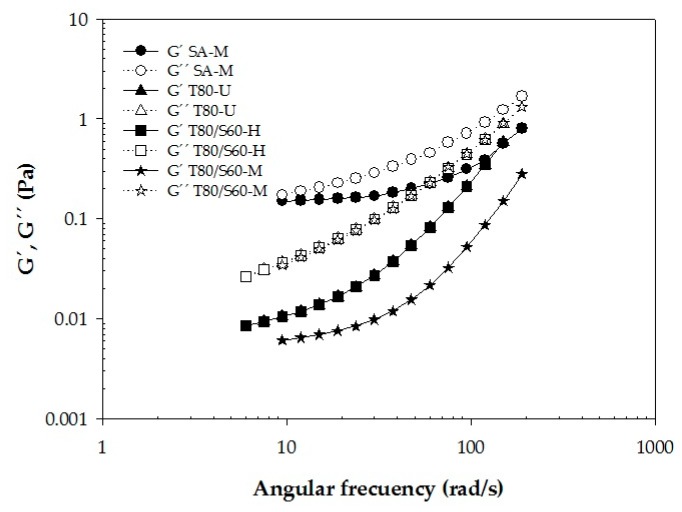
Oscillatory shear curves of beeswax–starch nanoemulsions. Filled symbols are G’: elastic module and empty symbols are G”: viscous module. Emulsifier type: SA, Stearic acid; T80, Tween 80; T80/S60, Tween 80/Span 60; shear process: H, homogenization; U, ultrasound; M, microfluidization.

Polysaccharide suspensions tend to be viscoelastic materials which can exhibit solid and liquid characteristics simultaneously [37]. The G′ and G′′ moduli refer to elastic and viscous responses of a given material, respectively [38]. Therefore, it is possible to quantify the predominance of either the solid or liquid character of a sample through dynamic measurements [39]. The non-Newtonian BW–SN samples were exposed to an amplitude oscillatory test, and exhibited a clear predominance of the viscous modulus (G′′ > G′), for a frequency range of 1–100 rad/s (Figure 7). This behavior could be the result of the structural configuration of BW–starch with the emulsifier, and of the potential effect of the emulsification process. 

### 2.5. Wettability

#### 2.5.1. $\gamma_{SV}$, $\gamma_{LV}$ and $\gamma_{SL}$

Edible coatings are capable of maintaining fresh produce quality and safety for a longer time, depending significantly on the BW–SN effectively spreading on the food surface that in turn is greatly influenced by its wettability. The equilibrium contact angles (*θ*) of the BW–SN were tested on the surface of tomato (Table 2), and ranged between 38.5° and 48.6°, which are <90°, implying that the BW–SN could adequately coat the tomato skin. The tomato surface is considered hydrophobic because it is covered with a wax layer comprising mainly naringenin–chalcone (52%), long-chain hydrocarbons (29%) and long-chain alcohols and triterpenols (19%) [40]. According to Sánchez-Ortega et al. [41], a higher contact angle (61.5°) was obtained for oleic acid–starch nanoemulsions added with T80 as emulsifier on the tomato surface. Choi et al. [42] reported higher contact angles between 87.5° and 90° for an emulsion of chitosan with T80 as emulsifier, tested on tangerine, melon, apple and tomato, indicating that this emulsion hardly coated the fruit’s skin. The $\gamma_{SV}$ of the tomato surface (Table 2) was 15.0 mN/m, the same as reported by Sánchez-Ortega et al. [41], and similar (17.4 mN/m) to that reported by Casariego et al. [43]. Low energy surfaces show $\gamma_{SV}$ < 100 mN/m, and they interact with liquids primarily through dipoles, induction, or hydrogen bonds [44,45]. The $\gamma_{LV}$ average values of the BW–SN (Table 2) varied from 27.9 mN/m for SA as emulsifier to 30.9 mN/m using T80/S60, in agreement with Sánchez-Ortega et al. [41] ($\gamma_{LV}$ = 30.9 mN/m) and Ramírez et al. [46], who reported $\gamma_{LV}$ of 34.3 mN/m for carboxymethylcellulose–sunflower oil and murta leaf extract on apple surface. 

#### 2.5.2. Wettability Coefficient

The wettability of the BW–SN is influenced by *W*a, which causes the liquid to spread on the solid flat surface, whereas *W*c brings about droplet shrinkage [43]. When *W*s < 0, the droplet minimizes the surface free energy, leading to partial wetting, and for *W*s = 0, the surface is totally wettable [47]. The emulsifier type showed a significant influence (*p* < 0.05) on *W*s of BW–SN (Table 3), whose average values were −7.5 ± 0.5 mN/m when using SA, −7.7 ± 1.0 mN/m for T80 and −7.2 ± 0.7 mN/m for T80/S60. These results were lower than those reported when chitosan–T80 was applied on tomato (−66.8 mN/m) and apple (−56.4 mN/m) surfaces [42]. The tomato surface contains natural waxes that confer a hydrophobic character [48], which is comprised of sterols, triterpenols, chalcones and alkanes, providing a smooth surface [40], and thus low *W*s results were obtained for all BW–SNs. 

### 2.6. Antimicrobial Effect 

**Figure 8:**
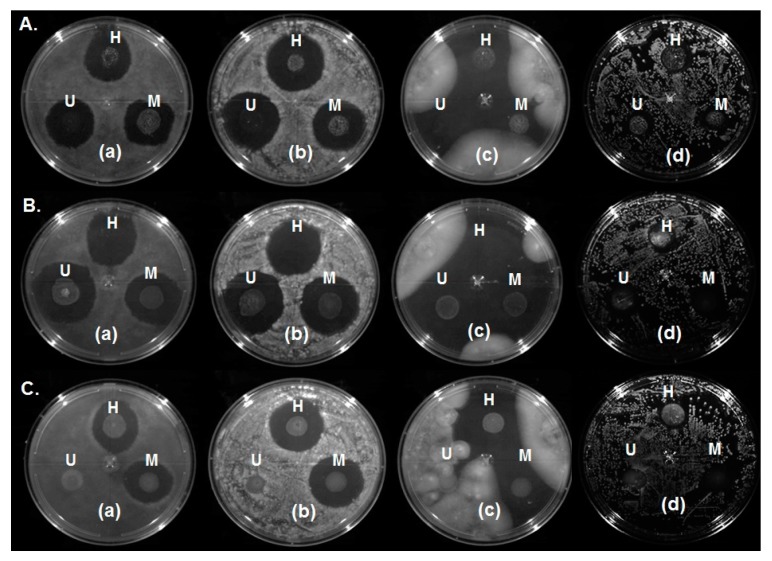
Antimicrobial effect of beeswax–starch nanoemulsion with (**A**) SA: stearic acid; (**B**) T80: Tween 80; and (**C**) T80/S60: Tween80/Span60 at different shear process against (**a**) *R. stolonifer*, (**b**) *C. gloeosporioides*, (**c**) *B. cinerea*, (**d**) *S.* Saintpaul. Emulsification process: U = ultrasound, H = homogenization, M = microfluidization.

Arredondo-Ochoa et al. [4] have reported the use of natural antimicrobial agents incorporated into a nanoemulsified matrix that were effective against microorganisms commonly found in fresh produce. In this study, the antimicrobial activity of the BW–SN was significantly influenced (*p* < 0.05) by the emulsifier type and emulsification process, in agreement with Liang et al. [33]. Inhibition zones of BW–SN using SA (Figure 8A) were 24.3 ± 2.4 mm for *R. stolonifer*, 28.7 ± 1.9 mm for *C. gloeosporioides*, 27.9 ± 8.0 mm for *B. cinerea* and 13.9 ± 0.7 mm for *S.* Saintpaul. For BW–SN using T80 (Figure 8B), inhibition zones were 28.9 ± 1.6 mm for *R. stolonifer*, 30.2 ± 1.3 mm for *C. gloeosporioides*, 34.7 ± 3.2 mm for *B. cinerea* and 14.5 ± 0.4 mm for *S.* Saintpaul. Additionally, BW–SN using the T80/S60 mixture (Figure 8C), either homogenized or microfluidized, exhibited inhibitory effects against *R. stolonifer* (28.0 ± 4.7 mm), *C. gloeosporioides* (27.5 ± 0.3 mm), *B. cinerea* (31.5 ± 1.7 mm) and *S.* Saintpaul (14.4 ± 0.5 mm). Similarly, Salvia-Trujillo et al. [31] reported inactivation of *Escherichia coli* with sodium alginate nanoemulsions incorporated with lemongrass or clove oil and T80 as emulsifier under a microfluidization process. On the other hand, ultrasonically produced BW–SN did not show an inhibitory effect against any of the tested microorganisms, indicating a significant (*p* < 0.05) interaction with the emulsifier type-emulsification process, which prevented antimicrobial activity. According to Teixeira et al. [49] and Lu et al. [24], the antimicrobial activity of nanoemulsions is favored by easier diffusion to microbial cell membrane from the nanosized droplets. In this sense, the highest particle size (1054.3 ± 4.0 nm) obtained for BW–SNs using T80/S60, and their interaction with the type of processing (ultrasound), may explain their antimicrobial activity inhibition. BW–SN added with T80 and microfluidized inhibited every one of the three fungi and the pathogenic bacteria tested, using a mixture of lauric arginate ester (LAE) (0.2% *w*/*w*), and natamycin (NAT) (0.04% *w*/*w*) [4].

## 3. Materials and Methods 

### 3.1. Chemicals

Commercial oxidized starch (OS) (carbonyl + carboxyl groups = 15.7 ± 1.2% *w*/*w*), and non-crystallizable sorbitol were provided by Ingredion (San Juan del Río, Qro., México). BW, stearic acid (SA), Tween 80 (T80), Span 60 (S60) and morpholine were supplied by Sigma (St. Louis, MO, USA). Lauric arginate ester (LAE) was acquired from Vedeqsa-Lamirsa (Terrassa, Barcelona, Spain) while natamycin (NAT) was purchased from EcoBio (Columbus, OH, USA).

### 3.2. Microorganisms

*Rhizopus stolonifer* (CDBB-H-318) and *Botrytis cinerea* (CDBB-H-1556) were provided by the national collection of microbial strains and cell cultures (CINVESTAV, CDMX, México), whereas *Colletotrichum gloeosporioides* strain was ATCC 42374. *Salmonella* Saintpaul S70 was supplied from the culture collection of the center for food research and development (CIAD, Hermosillo, Son., México).

### 3.3. Culture Media

Potato dextrose agar (PDA) (Bioxon, CDMX, México) was used to grow *R. stolonifer*, *C. gloeosporioides* and *B. cinerea*. Tryptone soy agar (TSA) (Bioxon) was used for *S.* Saintpaul growth. 

### 3.4. BW Thermal Analysis

The thermal behavior of BW was evaluated using a DSC (Q200 TA Instruments, New Castle, DE, USA) equipped with a refrigerated intracooling system. High-purity indium was used as standard and dry nitrogen as purge gas. Ten mg of BW was weighed into a hermetically sealed aluminum pan. The sample was equilibrated at 90 °C before being cooled to −40 °C at a rate of 10 °C/min, kept isothermally at −40 °C for 10 min, and reheated to 90 °C at 5 °C/min. Measurements were performed in triplicate and heat flow (W/g) was plotted against temperature (°C). Onset temperature and maximum peak (°C) were obtained from thermal curves of crystallization and melting, while enthalpy (J/g) was determined by integrating the area under each thermogram in the range 10–80 °C, using the Universal Analysis 2000 V 4.5 software (TA Instruments) [17]. 

### 3.5. BW–Starch Nanoemulsion (BW–SN) Formation

OS suspension (3% *w*/*w*) and non-crystallizable sorbitol (2.4% *w*/*w*) were heated at 85 °C for 20 min under magnetic stirring to complete starch gelatinization. Nanoemulsions formation was carried out following the reports of Hagenmaier and Baker [50]; Muscat et al. [51] and Santos et al. [52], with modifications. Briefly, molten BW (1% *w*/*w*) plus morpholine (0.15% *w*/*w*) as cosolvent were added with the selected emulsifier in varying ratios (*w*/*w*): BW:SA (5:1), BW:T80 (1:2.5) or BW:T80/S60 (1:2.5). The starch dispersion was kept at 85 °C along with a mixture of two antimicrobial agents (LAE (0.2% *w*/*w*) and NAT (0.04% *w*/*w*)) according to Arredondo-Ochoa et al. [4]. The primary emulsions were subjected to three different emulsification processes, homogenization using a high-speed mixer (IKA T25-Ultra-Turrax, Wilmington, DE, USA) at 21,500 rpm for 3 min; ultrasound for 5 min continuously, at 20 kHz frequency and 75% amplitude using a sonicator (VCX 500 Vibra-Cell, Newtown, CT, USA); or high-pressure processing employing a microfluidizer (M110P Nano DeBEE, Easton, MA, USA) at 150 MPa for 3 cycles [3,41]. In order to find the best conditions to obtain a stable antimicrobial emulsion with BW nanosized particles, a 3^2^ factorial design was conducted. Main factors were the type of emulsifier, and emulsification process, at three levels: three types of emulsifiers, and three emulsification processes, whereas the response variables were physicochemical, rheological, wettability and antimicrobial properties. The obtained BW–starch nanoemulsions were stored at 4 ± 1 °C for subsequent characterization.

### 3.6. Physicochemical Characterization of BW–SN

#### 3.6.1. Particle Size and PDI

The droplet size of BW–SN was determined using a Zetasizer Nano ZS (ZEN 3600 Malvern, Worcestershire, UK), by dynamic light scattering (DLS) technique working at 633 nm at 25 °C with a backscatter detector at 173°. Samples were previously diluted with ultra-pure water (1:20) to avoid multiple scattering effects. Size distribution curves as a function of intensity (%), average droplet size (z-average) (nm), and PDI were used to characterize the BW droplet dispersion in the emulsions [20]. 

#### 3.6.2. ζ-Potential

The lipid droplet surface charge (ζ–potential) of BW–SN was evaluated by measuring their electrophoretic mobility when an electric field was applied by phase-analysis light scattering (PALS) using a Zetasizer Nano ZS (Malvern). The Smoluchowski model was applied with the instrument’s software to obtain ζ–potential (mV) values, indicating potential stability of colloidal systems [25].

#### 3.6.3. pH

The pH was obtained from direct measurements of BW–SN with a pH meter (HI-2216 Hanna Instruments, Woonsocket, RI, USA).

#### 3.6.4. Color

The color of BW–SN was determined with a colorimeter (CR-400 Konica Minolta, Osaka, Japan), set up for illuminant D65 and 10° observer angle at room temperature, calibrated with a standard white plate. The Commission Internationale de lÉclairage (CIE) L*, a*, and b* values were determined and the whiteness index (WI) was calculated using Equation (1) [53]:

WI = 100 − [(100 − L)^2^ + a^2^ + b^2^]^0.5^ (1)

### 3.7. TEM

Nanoemulsions were observed by negative-staining electron microscopy as a direct measurement of droplet size and shape. The sample was adsorbed onto carbon-coated copper/palladium grids for 1 min, and then negatively stained by floating the grids face-down on a drop of 2% (*w*/*v*) ammonium molybdate, pH 6.5, for 1 min. The grids were observed using TEM (JEM-1010 JEOL, Peabody, MA, USA) at an acceleration voltage of 80 kV and the images was obtained using a digital micrograph 3.1 software (Gatan, Pleasanton, CA, USA) [23].

### 3.8. Rheological Behavior of BW–SN

Simple shear flow and oscillatory tests of BW–SN were conducted with a controlled stress rheometer (AR-G2, TA Instruments, New Castle, DE, USA), fitted with double-concentric cylinder geometry (21.96 mm outside diameter, 20.38 mm inside diameter, 59.50 mm height, and 500 µm gap from the base), at 25.0 ± 0.1 °C controlled by a circulating water bath (Cole Parmer Polystat and Peltier AR-G2). Estimation of viscosity (η) as a function of shear rate $(\dot{\gamma )}$ was performed in the range 1–300 s^−1^; whereas linear oscillatory shear flow was used to estimate viscoelastic properties (elastic modulus, G’; viscous modulus, G”) in a frequency range of 0.1–100 rad/s in the linear viscoelastic regime. Measurements were carried out in duplicate and experimental data were analyzed directly with the TA Rheology Advantage Data Analysis V.5.7.0 (TA Instruments, Crawley, UK) software [37].

### 3.9. Wettability of BW–SN

The wettability coefficient (*W*s) of a solid by a liquid is determined by the balance between adhesive forces (*W*a) of the liquid on the solid (Equation (2)), and cohesive forces (*W*c) of the liquid (Equation (3)) expressed in mN/m, which takes into account the action of three surface forces (Equation (4)) [42]:

(2) $$Wa=(\gamma_{LV}+\gamma_{SV}-\gamma_{SL}),$$

(3) $$Wc=2\;\gamma_{LV},$$

(4) $$Ws=Wa-Wc=(\gamma_{LV}+\gamma_{SV}-\gamma_{SL})-2\;\gamma_{LV}=\gamma_{SV}+\gamma_{SL}-\gamma_{LV},$$

where: $\gamma_{LV}$ = liquid–vapor surface tension (mN/m), $\gamma_{SV}$ = solid–vapor interfacial tension (mN/m), $\gamma_{SL}$ = solid–liquid interfacial tension (mN/m).

#### 3.9.1. Solid–Vapor Surface Tension ($\gamma_{SV})$

The $\gamma_{SV}$, also known as critical surface tension, was calculated according to the Zisman [44] method based on contact angle (*θ*), using a drop shape analyzer DSA30 (KRUSS, Hamburg, Germany), fitted with DSA4 software. A drop (9 µL) of reference liquid (ethylene glycol, formamide, glycerol, methanol, propylene glycol, and deionized water), with known surface tension, was gently dispensed on horizontal tomato surface for contact time of 60 s. Finally, a Zisman plot of the surface tension of each liquid versus its corresponding cos (*θ*) was carried out, and extrapolation of the curve when cos (*θ*) = 1 corresponded to the value of $\gamma_{SV}$ [47].

#### 3.9.2. Liquid–Vapor Surface Tension ($\gamma_{LV}$)

The $\gamma_{LV}$ of BW–SN was measured using a surface tensiometer fitted with a 6 cm-diameter platinum ring (CSC Scientific, Fairfax, VA, USA) according to the Du Nouy ring method [54]. A correction factor (P/[D − d]) proposed by Zuidema & Waters [55] was introduced, where P is apparent surface tension, D is water density and d is air density at 25 °C.

#### 3.9.3. Solid–Liquid Surface Tension ($\gamma_{SL}$)

The $\gamma_{SL}$ was calculated from the Young’s equation (Equation (5)), considering the two surface forces previously estimated. The contact angle (*θ*) formed by each BW–SN on the tomato surface was measured according to Section 3.9.1 [46].

(5) $$\cos\theta =(\gamma_{SV}-\gamma_{SL})/\gamma_{LV}$$

### 3.10. Antimicrobial Activity of BW–SN

Antimicrobial activity of BW–SN was evaluated against three deteriorative fungi (*R. stolonifer*, *C. gloeosporioides* and *B. cinerea*) and a pathogenic bacterium (*S.* Saintpaul S70) commonly present in fresh produce according to Arredondo-Ochoa et al. [4]. The antimicrobial challenge was conducted contacting 20 µL of each nanoemulsion with 10^5^ CFU/mL of tested fungi in PDA plates, followed by incubation at 28 ± 1 °C for 24 h. In addition, TSA plates similarly inoculated with the pathogenic bacterium were incubated at 37 ± 1 °C for 24 h. The antimicrobial effect was determined by the inhibition zone diameter (mm) [56].

### 3.11. Statistical Analysis

Physicochemical, rheological, wettability, and antimicrobial properties of nanoemulsions were conducted in triplicate, and the Tukey test was used to determine significant difference (*p* < 0.05) among mean values, employing the JMP statistical software, version 5.0.1 (Cary, NC, USA). 

## 4. Conclusions

BW–SNs were successfully produced and a significant influence of emulsifier type and nanoemulsification process was evidenced from the physicochemical, rheological and antimicrobial properties. Using T80 and microfluidization, BW–SNs of low particle size were produced, suggesting homogeneous distribution of ingredients leading to stability and translucency, showing a Newtonian behavior, and efficient wettability properties. This BW–SN led to complete inhibition of deteriorative fungi and a pathogenic bacterium that are commonly present in fresh produce, making it useful as a suspension able to produce edible coatings to protect and preserve fresh food products.

## Figures and Tables

**Table 1:** Average droplet size (z-average) and polydispersity index (PDI) of BW–starch nanoemulsions.

Emulsifier	Process	z-Average (nm)	PDI	pH
SA	Homogenization	244.8 ± 2.1 ^e^	0.7 ± 0.2 ^a,b^	8.3 ± 0.0 ^c^
	Ultrasound	515.1 ± 5.9 ^d^	0.5 ± 0.2 ^c,d^	8.3 ± 0.0 ^c^
	Microfluidization	256.8 ± 3.9 ^e^	0.6 ± 0.1 ^c,d^	8.3 ± 0.0 ^c^
T80	Homogenization	185.5 ± 8.5 ^f^	0.3 ± 0.0 ^b,c,d^	8.6 ± 0.0 ^a^
	Ultrasound	111.9 ± 5.6 ^g^	0.4 ± 0.0 ^a,b,c^	8.6 ± 0.1 ^a^
	Microfluidization	77.7 ± 6.2 ^h^	0.3 ± 0.0 ^d^	8.6 ± 0.0 ^a^
T80/S60	Homogenization	913.8 ± 10.6 ^b^	0.8 ± 0.1 ^a,b^	8.4 ± 0.0 ^b^
	Ultrasound	1054.3 ± 4.0 ^a^	0.7 ± 0.0 ^a,b^	8.4 ± 0.1 ^b^
	Microfluidization	891.9 ± 8.9 ^c^	0.8 ± 0.2 ^a^	8.3 ± 0.1 ^b^

SA: stearic acid; T80: Tween 80; S60: Span 60. Data are the mean ± standard deviation. Superscript letters a–h are used next to reported values, indicating that if the same letter appears in the same column, the values compared are not significantly different (*p* > 0.05).

**Table 2:** Contact angle and surface tensions of BW–starch nanoemulsions on the tomato surface.

Emulsifier	Process	Contact Angle (*θ*)	Cos *θ*	$\gamma_{SV}$ (mN/m)	$\gamma_{LV}$ (mN/m)	$\gamma_{SL}$ (mN/m)
SA	Homogenization	45.8	0.7	15.0	26.0	−3.1
	Ultrasound	43.1	0.7	15.0	28.0	−5.4
	Microfluidization	40.0	0.8	15.0	29.7	−7.7
T80	Homogenization	42.2	0.7	15.0	26.6	−4.6
	Ultrasound	44.1	0.7	15.0	25.8	−3.4
	Microfluidization	48.6	0.7	15.0	26.3	−2.4
T80/S60	Homogenization	41.1	0.8	15.0	31.4	−8.5
	Ultrasound	38.5	0.8	15.0	30.0	−8.4
	Microfluidization	40.2	0.7	15.0	31.4	−8.9

SA: Stearic acid; T80: Tween 80; S60: Span 60.

**Table 3:** Adhesive forces (*W*a), cohesive forces (*W*c) and wettability coefficient (*W*s) of BW–starch nanoemulsions.

Emulsifier	Process	*W*a (mN/m)	*W*c (mN/m)	*W*s (mN/m)
SA	Homogenization	44.2 ± 1.2 ^b^	52.1 ± 1.7 ^b^	−7.9 ± 1.1 ^a^
	Ultrasound	48.4 ± 1.8 ^b^	56.0 ± 1.9 ^b^	−7.6 ± 1.3 ^a^
	Microfluidization	52.4 ± 1.7 ^b^	59.4 ± 1.0 ^b^	−7.0 ± 1.6 ^a^
T80	Homogenization	46.2 ± 2.1 ^c^	53.2 ± 1.0 ^c^	−7.0 ± 2.0 ^a^
	Ultrasound	44.2 ± 2.0 ^c^	51.5 ± 1.9 ^c^	−7.3 ± 0.6 ^a^
	Microfluidization	43.7 ± 3.3 ^c^	52.6 ± 1.9 ^c^	−8.9 ± 1.6 ^a^
T80/S60	Homogenization	54.9 ± 0.3 ^a^	62.7 ± 1.0 ^a^	−7.8 ± 1.2 ^a^
	Ultrasound	53.4 ± 1.1 ^a^	59.9 ± 1.0 ^a^	−6.5 ± 0.5 ^a^
	Microfluidization	55.3 ± 4.3 ^a^	62.7 ± 2.6 ^a^	−7.5 ± 2.2 ^a^

SA: stearic acid; T80: Tween 80; S60: Span 60. Data are the mean ± standard deviation. Superscript letters a–c are used next to reported values, indicating that if the same letter appears in the same column, the values compared are not significantly different (*p* > 0.05).

## Abbreviations

BW	Beeswax
OS	Oxidized starch
SN	Starch nanoemulsions
SA	Stearic acid
T80	Tween 80
S60	Span 60
LAE	Lauric arginate ester
NAT	Natamycin
PDA	Potato dextrose agar
TSA	Tryptone soy agar
DSC	Differential scanning calorimeter
DLS	Dynamic light scattering
PALS	Phase-analysis light scattering
PDI	Polydispersity index
WI	Whiteness index
TEM	Transmission electron microscopy
*W*s	Wettability coefficient
*W*a	Adhesive forces
*W*c	Cohesive forces
T_oc_	Crystallization onset temperature
T_c_	Maximum crystallization peak
T_om_	Melting onset temperature
T_m_	Maximum melting peak

## References

[1] Cushen M., Kerry J., Morris M., Cruz-Romero M., Cummins E. (2012). Nanotechnologies in the food industry-recent developments, risks and regulation. Trends Food Sci. Technol..

[2] Momin J.K., Joshi B.H., Rai M., Ribeiro C., Mattoso L., Duran N. (2015). Nanotechnology in Foods. Nanotechnologies in Food and Agriculture.

[3] Salvia-Trujillo L., Rojas-Graü A., Soliva-Fortuny R., Martín-Belloso O. (2015). Use of antimicrobial nanoemulsions as edible coatings: Impact on safety and quality attributes of fresh-cut *Fuji* apples. Postharvest Biol. Technol..

[4] Arredondo-Ochoa T., García-Almendárez B.E., Amaro-Reyes A., Rivera-Pastrana D.M., Gutiérrez-López G.F., Martín-Belloso O., Regalado-Gonzalez C. (2017). Design and characterization of corn starch edible films including beeswax and natural antimicrobials. Food Bioprocess Technol..

[5] Sagalowicz L., Leser M.E. (2010). Delivery systems for liquid food products. Curr. Opin. Colloid Interface Sci..

[6] Gutiérrez J.M., González C., Maestro A., Solè I., Pey C.M., Nolla J. (2008). Nano-emulsions: New applications and optimization of their preparation. Curr. Opin. Colloid Interface Sci..

[7] Qian C., McClements D.J. (2011). Formation of nanoemulsions stabilized by model food-grade emulsifiers using high-pressure homogenization: Factors affecting particle size. Food Hydrocoll..

[8] Donsì F., Annunziata M., Sessa M., Ferrari G. (2011). Nanoencapsulation of essential oils enhance their antimicrobial activity in foods. LWT-Food Sci. Technol..

[9] Delmas T., Piraux H., Couffin A.C., Texier I., Vinet F., Poulin P., Cates M.E., Bibette J. (2011). How to prepare and stabilize very small nanoemulsions. Langmuir.

[10] McClements D.J. (2016). Context and Background. Food Emulsions: Principles, Practices, and Techniques.

[11] Mason T.G., Wilking J.N., Meleson K., Chang C.B., Graves S.M. (2006). Nanoemulsions: Formation, structure, and physical properties. Phys. Condens. Matter.

[12] McClements D.J., Rao J. (2011). Food-grade nanoemulsions: Formulation, fabrication, properties, performance, biological fate, and potential toxicity. Crit. Rev. Food Sci. Nutr..

[13] Kralova I., Sjöblom J. (2009). Surfactants used in food industry: A review. J. Dispers. Sci. Technol..

[14] Silva H.D., Cerqueria M.A., Vicente A.A. (2012). Nanoemulsion for food applications: Development and characterization. Food Bioprocess Technol..

[15] Attama A.A., Shicke B.C., Müller-Goymann C.C. (2006). Further characterization of theobroma oil-beeswax admixtures as lipid matrices for improved drug delivery systems. Eur. J. Pharm. Biopharm..

[16] Aichholz R., Lorbeer E. (1999). Investigation of combwax of honeybees with high-temperature gas chromatography and high-temperature gas chromatography-chemical ionization mass spectrometry. I. High-temperature gas chromatography. J. Chromatogr. A.

[17] Buchwald R., Breed M.D., Greenberg A.R. (2008). The thermal properties of beeswaxes: Unexpected findings. J. Exp. Biol..

[18] Yilmaz E., Ögütcü M. (2014). Properties and stability of hazelnut oil organogels with beeswax and monoglyeride. J. Am. Oil Chem. Soc..

[19] Brar S.K., Verma M. (2011). Measurement of nanoparticles by light-scattering techniques. Trends Anal. Chem..

[20] Kaszuba M., McKnight D., Connah M.T., McNeil-Watson F.K., Nobbmann U. (2008). Measuring sub nanometer sizes using dynamic light scattering. J. Nanopart. Res..

[21] Rao J., McClements D.J. (2012). Food-grade microemulsions and nanoemulsions: Role of oil phase composition on formation and stability. Food Hydrocoll..

[22] Ghosh V., Mukherjee A., Chandrasekaran N. (2013). Ultrasonic emulsification of food-grade nanoemulsion formulation and evaluation of its bactericidal activity. Ultrason. Sonochem..

[23] Salvia-Trujillo L., Rojas-Graü A., Soliva-Fortuny R., Martín-Belloso O. (2013). Physicochemical characterization of lemongrass essential oil-alginate nanoemulsions: Effect of ultrasound processing parameters. Food Bioprocess Technol..

[24] Lu W., Zhang Y., Tan Y.Z., Hu K.L., Jiang X.G., Fu S.K. (2005). Cationic albumin-conjugated pegylated nanoparticles as novel drug carrier for brain delivery. J. Control. Release.

[25] Kaszuba M., Corbett J., Watson F.M., Jones A. (2010). High-concentration zeta potential measurements using light-scattering techniques. Philos. Trans. R. Soc. A.

[26] Guerra-Rosas M.I., Morales-Castro J., Ochoa-Martínez L.A., Salvia-Trujillo L., Martín-Belloso O. (2016). Long-term stability of food-grade nanoemulsions from high methoxyl pectin containing essential oils. Food Hydrocoll..

[27] Chanamai R., McClements D.J. (2001). Depletion flocculation of beverage emulsions by gum arabic and modified starch. J. Food Sci..

[28] McClements D.J., Xiao H. (2012). Potential biological fate of ingested nanoemulsions: Influence of particle characteristics. Food Funct..

[29] Mayer S., Weiss J., McClements D.J. (2013). Behavior of vitamin E acetate delivery systems under simulated gastrointestinal conditions: Lipid digestion and bioaccessibility of low-energy nanoemulsions. J. Colloid Interface Sci..

[30] Guzey D., McClements D.J. (2006). Formation, stability and properties of multilayer emulsions for application in the food industry. Adv. Colloid Interface Sci..

[31] Salvia-Trujillo L., Rojas-Graü A., Soliva-Fortuny R., Martín-Belloso O. (2014). Physicochemical characterization and antimicrobial activity of food-grade emulsions and nanoemulsions incorporating essential oils. Food Hydrocoll..

[32] Klang V., Matsko N.B., Valenta C., Hofer F. (2011). Electron microscopy of nanoemulsions: An essential tool for characterisation and stability assessment. Micron.

[33] Liang R., Xu S., Shoemaker C.F., Li Y., Zhong F., Huang Q. (2012). Physical and antimicrobial properties of peppermint oil nanoemulsions. J. Agric. Food Chem..

[34] Hopkins E.J., Chang C., Lam R.S.H., Nickerson M.T. (2015). Effects of flaxseed oil concentration on the performance of soy protein isolate-based emulsion-type film. Food Res. Int..

[35] Rezvani E., Schleining G., Sümen G., Taherian A.R. (2013). Assessment of physical and mechanical properties of sodium caseinate and stearic acid based film-forming emulsions and edible films. J. Food Eng..

[36] Fabra M.J., Jiménez A., Atarés L., Talens P., Chiralt A. (2009). Effect of fatty acids and beeswax addition on properties of sodium caseinate dispersions and films. Biomacromolecules.

[37] Medina-Torres L., Santiago-Adame R., Calderas F., Gallegos-Infante J.A., González-Laredo R.F., Rocha-Guzmán N.E., Núñez-Ramírez D.M., Bernad-Bernad M.J., Manero O. (2016). Microencapsulation by spray drying of laurel infusions (*Litsea glaucescens*) with maltodextrin. Ind. Crops Prod..

[38] Bourbon A.I., Pinheiro A.C., Ribeiro C., Miranda C., Maia J.M., Teixeira J.A., Vicente A.A. (2010). Characterization of galactomannans extracted from seeds of *Gleditsia triacanthos* and *Sophora japonica* through shear and extensional rheology: Comparison with guar gum and locust bean gum. Food Hydrocoll..

[39] Campestrini L.H., Silveira J.L.M., Duarte M.E.R., Koop H.S., Noseda M.D. (2013). NMR and rheological study of *Aloe barbadensis* partially acetylated glucomannan. Carbohydr. Polym..

[40] Bauer S., Schulte E., Thier H.P. (2004). Composition of the surface wax from tomatoes. I. Identification of the components by GC/MS. Eur. Food Res. Technol..

[41] Sánchez-Ortega I., García-Almendárez B.E., Santos-López E.M., Reyes-González L.R., Regalado C. (2016). Characterization and antimicrobial effect of starch-based edible coating suspensions. Food Hydrocoll..

[42] Choi W.Y., Park H.J., Ahn D.J., Lee J., Lee C.Y. (2002). Wettability of chitosan coating solution on “*fuji*” apple skin. J. Food Sci..

[43] Casariego A., Souza B.W.S., Vicente A.A., Teixeira J.A., Cruz L., Díaz R. (2008). Chitosan coating surface properties as affected by plasticizer, surfactant and polymer concentrations in relation to the surface properties of tomato and carrot. Food Hydrocoll..

[44] Zisman W.A., Fowkes F.M. (1964). Relation of the equilibrium contact angle to liquid and solid constitution. Contact Angle, Wettability, and Adhesion.

[45] Cerqueira M.A., Lima A.M., Teixeira J.A., Moreira R.A., Vicente A.A. (2009). Suitability of novel galactomannans as edible coatings for tropical fruits. J. Food Eng..

[46] Ramírez C., Gallegos I., Ihl M., Bifani V. (2012). Study of contact angle, wettability and water vapor permeability in carboxymethylcellulose (CMC) based film with murta leaves (*Ugni molinae* Turcz) extract. J. Food Eng..

[47] Skurtys O., Velásquez P., Henriquez O., Matiacevich S., Enrione J., Osorio E.F. (2011). Wetting behavior of chitosan solutions on blueberry epicarp with or without epicuticular waxes. LWT-Food Sci. Technol..

[48] Jetter R., Kunst L., Samuels A.L., Riederer M., Müller C. (2006). Composition of plant cuticular waxes. Biology of the Plant Cuticle.

[49] Teixeira P.C., Leite G.M., Domingues R.J., Silva J., Gibbs P.A., Ferreira J.P. (2007). Antimicrobial effects of a microemulsion and a nanoemulsion on enteric and other pathogens and biofilms. Int. J. Food Microbiol..

[50] Hagenmaier R.D., Baker R.A. (1994). Wax microemulsions and emulsions as citrus coatings. J. Agric. Food Chem..

[51] Muscat D., Adhikari R., McKnight S., Guo Q., Adhikari B. (2013). The physicochemical characteristics and hydrophobicity of high amylose starch-glycerol films in the presence of three natural waxes. J. Food Eng..

[52] Santos T.M., Pinto A.M.B., de Oliveira A.V., Ribeiro H.L., Caceres C.A., Ito E.N., Azeredo H.M. (2014). Physical properties of cassava-starch-carnauba wax emulsion films as affected by component proportions. Int. J. Food Sci. Technol..

[53] Vargas M., Cháfer M., Albors A., Chiralt A., González-Martínez C. (2008). Physicochemical and sensory characteristics of yogurt produced from mixtures of cows’ and goats’ milk. Int. Dairy J..

[54] Harkins W.D., Jordan H.F. (1930). A method for the determination of surface and interfacial tension from the maximum pull on a ring. J. Am. Chem. Soc..

[55] Zuidema H.H., Waters G.W. (1941). Ring method for the determination of interfacial tension. Ind. Eng. Chem. Res..

[56] Zahid N., Ali A., Manickam S., Siddiqui Y., Maqbool M. (2012). Potential of chitosan-loaded nanoemulsions to control different *Colletotrichum* spp. and maintain quality of tropical fruits during cold storage. J. Appl. Microbiol..

